# Examining Drug-Resistant Tuberculosis Stigma Among Health Care Workers Toward the Development of a Stigma-Reduction Intervention: Protocol for a Scoping Review

**DOI:** 10.2196/43084

**Published:** 2023-01-13

**Authors:** Lolita Liboon Aranas, Khorshed Alam, Prajwal Gyawali, Rashidul Alam Mahumud

**Affiliations:** 1 Graduate Research Studies University of Southern Queensland Toowoomba Australia; 2 Graduate Studies Jose Rizal University Mandaluyong City Philippines; 3 School of Business University of Southern Queensland Toowoomba, Queensland Australia; 4 School of Health and Medical Sciences University of Southern Queensland Toowoomba, Queensland Australia; 5 National Health and Medical Research Council Clinical Trial Centre Faculty of Medicine and Health The University of Sydney Sydney Australia

**Keywords:** drug-resistant tuberculosis, health workers, stigma, tuberculosis stigma, TB stigma, DRTB stigma

## Abstract

**Background:**

Drug-resistant tuberculosis (DRTB) is an increasing threat to human health and economic security worldwide. Exacerbating the severity of DRTB is the low rate of service delivery, leading to increased community transmission of the disease, further amplified by stigma. Health workers are on the front line of service delivery; their efforts in all areas of disease control are suspected of having resulted in stigmatization, impacting patient-centered care. As a growing concern, attention to addressing the DRTB stigma confronting health workers is required. However, little is known about stigma among health workers delivering services to patients with DRTB. This scoping review will provide an overview that could help inform appropriate responses toward stigma-reduction interventions for these health workers.

**Objective:**

This scoping review protocol articulates a methodology that will examine the facets of DRTB-related stigma confronting health workers in high TB- and DRTB-burdened countries. This scoping review will (1) summarize stigma barriers and facilitators contributing to stigmatization among health workers delivering services to patients with DRTB, (2) identify the most common stigma barrier and facilitator, and (3) summarize the stigma-reduction intervention recommendations in the studies.

**Methods:**

Guided by Arksey and O’Malley’s framework and the recommendations of Munn et al, we will conduct a scoping review of relevant literature providing evidence of DRTB-related stigma among health workers from countries with a high burden of tuberculosis (TB) and DRTB. We will search published articles written in English from 2010 onward in electronic databases using Medical Subject Headings and keywords. Our search will apply a 3-step search strategy and use software tools to manage references and facilitate the entire scoping review process. The findings of our review will be presented following the Preferred Reporting Items for Systematic Reviews and Meta-Analyses for Scoping Reviews checklist. Our study is registered with Open Science Framework Registries.

**Results:**

This scoping review is part of a bigger project that will critically investigate stigma among health workers delivering services to patients resistant to TB medications. This study began in November 2021 and is expected to finish in 2023. The study has retrieved 593 abstracts out of 12,138 articles searched since February 2022 from the identified databases. The findings of this study will be published in a peer-reviewed journal.

**Conclusions:**

This review will provide an outline of the aspects of DRTB-related stigma confronting health workers. The findings of this review could help inform appropriate responses toward stigma-reduction interventions for these health workers. This is significant because interventions addressing related TB (and DRTB) stigma in the workplace are lacking.

**International Registered Report Identifier (IRRID):**

DERR1-10.2196/43084

## Introduction

### Background

Tuberculosis (TB) exists in every part of the world. Approximately 25% of the world’s population has latent TB and is at risk of developing the disease during their lifetime [1]. Accordingly, about 10 million people fall sick with this disease each year, two-thirds of whom are in Bangladesh, China, India, Indonesia, Nigeria, the Philippines, and South Africa [1]. Meanwhile, drug-resistant tuberculosis (DRTB) is the highest in China, India, and Russia, while 9 of the 30 countries with the highest DRTB burden are within the European region [1].

With over a million people becoming ill with DRTB each year, the disease is considered a significant contributor to drug resistance worldwide [2]. In 2021, overall, 3.6% of new TB cases and 18% of previously treated patients were resistant to at least 1 anti-TB drug [3]. However, DRTB has become more challenging as the number of people previously treated for the disease had declined because of COVID-19 pandemic–related disruptions to TB service delivery. In 2021, the burden of DRTB increased by 3%, with 450,000 new cases [3]. As the increasing incidence of DRTB burdens public health, the country’s economic security is threatened. Treatment of DRTB is prolonged and requires new-generation drugs, making it financially burdensome [4]. Additionally, the DRTB treatment success rate is lower than for new TB cases.

Ending the TB epidemic by 2035 is one of the priority goals of global health organizations. However, attaining this goal requires an urgent action that will accelerate efforts to diagnose, treat, and prevent TB (including DRTB). Such efforts can be achieved strategically by focusing on cutting-edge research and innovation, improving drug resistance surveillance, and strengthening public-private partnerships [3]. In the community, essential services such as case finding, diagnosis, treatment, health promotion and education, and psychosocial support are among the current strategies used to reduce the disease burden [1]. Central to these activities are the health workers, where DRTB essential services are integrated into community health services and directed toward patient-centered care [5]. Patient-centered care involves clinical service delivery and provides support for the patient’s social and economic conditions that increase the burden of DRTB, such as malnutrition, poor housing, and financial and geographic barriers to health care access. In addition, it provides a holistic approach by incentivizing patients, treatment supporters, and health care providers [6].

The burden of DRTB is a multifaceted health challenge and social issue [6,7]. Medications for DRTB are more toxic and expensive [8], and the risks and treatment outcomes are influenced by various determinants and are commonly associated with stigma [9,10]. Stigma is the negative evaluation of oneself tainted by a particular attribute, making one constantly unsure how others will identify or receive it [11]. In terms of stigma in health, patients and communities distinguish and label various health conditions, views, and perceptions differently. As such, stigma influences community norms, interpersonal relations, and health institutions’ culture [12,13].

A growing area of research has evidenced the stigma associated with DRTB. Datiko et al [14] posited that stigmatization of TB (including DRTB) affects prevention, care, and treatment. As a result, TB-related stigma contributes to the increasing DRTB burden and is a crucial predictor of its high incidence [15], and thus, warrants reduction interventions [9]. Tackling DRTB stigma is significant because it affects the quality of life of people affected by the disease and, in part, confronts health workers [16-18]. In health care, for health workers delivering essential services, the stigma surrounding TB (in general) is commonly associated with the “dirty work” stigma [19]. Hughes (1962) referred to dirty work as tasks and occupations perceived by the community as disgusting or degrading.

Current literature reviews on TB-related stigma suggest that most studies are geared toward understanding stigma among patients and their families. For example, in their qualitative review, Juniarti and Evans [20] explored stigma and the impact of either having TB, or a family member having TB. Craig et al [21] mapped TB stigma research and found that the majority of studies aimed to assess the knowledge, attitudes, and beliefs regarding TB (including DRTB) in low-incidence countries. The review of Sommerland et al [22] evaluated TB stigma–reduction interventions within the community. Notably, their reviews generally highlight the impact of TB stigma on individuals and communities. However, the growing evidence of DRTB-related stigma among health workers requires attention. There is a need to better understand the stigma surrounding the disease and how it is currently addressed in health facilities.

We conducted a preliminary search of MEDLINE, the Cochrane Database of Systematic Reviews, and Joanna Briggs Institute (JBI) Evidence Synthesis and found no current systematic reviews or scoping reviews underway on DRTB-related stigma among health workers. Using Arksey and O’Malley’s [23] methodological framework and the recommendations of Munn et al [24], we will conduct a scoping study to examine the literature about the stigma of health workers delivering services for DRTB, explicitly examining the facets of disease stigma. Stangl et al [25] mentioned that the facets of stigmatization primarily constitute drivers and facilitators (eg, government policies, institutional support, and exaggerated fear) that influence disease outcomes among affected populations, organizations, and institutions. This scoping review will provide an overview of the aspects of DRTB-related stigma confronting health workers and other relevant information. The result of this review could help inform appropriate responses toward stigma-reduction interventions for these health workers. This is significant because interventions addressing related TB (and DRTB) stigma in the workplace are lacking [9]. Nyblade et al [26][26] stated that it is crucial to sustainably address this issue, from national to facility levels, because it undermines the delivery of quality health care and successful health outcomes.

### Review Question

Based on our objective, the overarching research question in this review is: “What aspects of DRTB-related stigma are confronting health workers in high TB- and DRTB-burden countries?” We will consider subquestions:

- What are the stigma drivers confronting the health workers, and what is the most common stigma driver?
- What are the stigma facilitators confronting the health workers, and what is the most common stigma facilitator?
- What stigma-reduction interventions are recommended in the study?

## Methods

We will apply the JBI scoping review methodology and use the features and functionalities of the JBI System for the Unified Management, Assessment, and Review of Information (SUMARI) [27] web-based software tool throughout the review process. The scoping review is registered with OSF Registries.

### Inclusion and Exclusion Criteria

Our review will apply the Participants/Concept/Context criteria recommended by Peter et al [28]. The eligibility criteria for this review will be as follows.

#### Participants

The participants in the study are health workers, including physicians, nurses, midwives, medical technologies, pharmacists, and other allied professionals in health care settings such as hospitals, clinics, community centers, and TB treatment facilities delivering DRTB services such as case-finding, screening, diagnosis, treatment, and prevention.

#### Concept

This review will include evidence of the stigma of health workers delivering services to patients with DRTB. Concepts to be examined are stigma drivers and facilitators, including but not limited to beliefs, fears, lack of awareness about the DRTB and stigma, inability to clinically manage the condition, negative attitudes, and institutionalized procedures or practices.

#### Context

This review will consider available data from countries identified in the World Health Organization’s list of high TB- and DRTB-burden countries (Table S1 in Multimedia Appendix 1). Any article whose study participants are not included in the list will not be considered. To be eligible, stigma confronts the health worker delivering services to patients with DRTB. Studies in which the context of stigma relates to drug-susceptible TB (DSTB), DRTB patients, and their families will be excluded from the review. We will also exclude sources coming from opinion articles, commentaries, or editorial reviews.

### Types of Sources

We will consider qualitative, quantitative, and mixed study designs, including descriptive observational studies, case reports, and gray literature on stigma confronting health workers delivering essential services to patients with DRTB. Peters et al [29] noted that one crucial point in scoping reviews is to draw upon data from any source of evidence and research methodology; thus, identifying articles for a scoping review will be less restrictive. As a result, an appraisal of the methodological quality of the available evidence on health workers’ stigma in this scoping review will not be performed. As mentioned, we could not find any systematic or scoping reviews on this topic. However, if any review is found as a result of a rigorous literature search in this study, those that meet the inclusion criteria will also be considered, depending on the research question.

### Search Strategy

This review will locate published articles using the 3-step search strategy recommended by the JBI scoping review guidelines [28]. We first conducted a pilot search of the PubMed and EBSCO databases to identify articles on the topic using Medical Subject Headings terms and keywords. In the next step, we will create a search protocol using the identified text terms in the titles, abstracts, and keywords and use the protocol to develop a full search strategy for CINAHL and MEDLINE (Table S2 in Multimedia Appendix 1). To enhance our search strategy, we will seek expert advice from university librarians to develop a search protocol that will be used to retrieve potentially relevant articles from the databases. The search strategy will be applied to Cochrane, ProQuest, Scopus, Web of Science, and other databases and information sources. Finally, we will search for gray literature and additional resources in electronic sources such as Google Scholar, ProQuest Dissertation, Open Access Theses and Dissertations, Networked Digital Library of Theses and Dissertations, and researchgate.net.

### Study Selection

After the initial search, all identified citations will be collated and uploaded to EndNote X9, de-duplicated, and imported to JBI SUMARI. Two independent reviewers will subsequently screen the titles and abstracts to assess the inclusion criteria for the review. We will retrieve potentially relevant sources for full-text screening to confirm the eligibility of the study for analysis. Any study that includes stigma associated with DRTB and health workers will qualify for the analysis. The bibliographies of studies meeting the inclusion criteria will be scanned to identify additional articles eligible for inclusion. Any disagreements between the reviewers at each stage of the selection process will be resolved through discussion or with an additional reviewer. The scoping review will record and report reasons for excluding sources of evidence in the full text that do not meet the inclusion criteria.

### Data Charting

We developed a draft extraction chart (Table S3 in Multimedia Appendix 1) that will be piloted to examine results from 3 to 5 articles. The piloting is aimed to ensure that the extraction chart captures all relevant information to satisfy the scoping review objectives and questions. The data extraction chart includes specific details about the participants, concept, context, study methods, and key findings relevant to the review questions. Henceforth, to ensure comprehensive coverage in the literature search, the search process will be iterative and require reviewers to engage reflexively [23]. We will have an opportunity to modify and revise the data extraction chart at this stage to suit our needs. Once all the reviewing team members are satisfied with the pilot charting, the 2 independent reviewers will use the finalized chart to extract relevant data from the identified studies. The reviewers will detail the modifications in the scoping review. If appropriate, we will contact the authors of papers to request missing or additional data, where required.

### Data Analysis and Presentation

We will use the Preferred Reporting Items for Systematic Reviews and Meta-analyses extension for scoping review flow diagram [30] to report the review search and inclusion pathway. First, the details of the included articles will be summarized and presented in tabular form outlining the authors, year of publication, settings, outcome measures, and main descriptions of the results to provide an overview of the extent, nature, and distribution of the studies in this scoping review. Second, we will present a summary list of findings with illustrations and narratives. Third, we will thematically synthesize the key findings of the studies and will present a graphic diagram following the aspects of DRTB stigma (stigma drivers and facilitators) identified in the research questions of this scoping review. Lastly, we will provide a summary of our findings and describe how the results relate to the objectives and questions of this scoping review.

## Results

This study commenced in October 2021. After conception, this scoping protocol was registered in OSF to promote transparency in our research and prevent duplication by others. The protocol refinement was completed in December 2021, and soon after, a pilot search of the PubMed and EBSCO databases was performed to inform the search strategy’s development. The study has retrieved 593 abstracts out of 12,138 articles searched since February 2022 from the identified databases. This study is expected to finish in 2023. The findings of this study will be published in a peer-reviewed journal.

## Discussion

### Principal Findings

The stigma surrounding DRTB is a growing concern. Research has shown that frontline health care workers delivering DRTB services are affected by such stigma, so it requires attention [9]. In scanning the literature, gaps emerged as most interventions were geared toward patients with DRTB and their families, less toward health workers. Also of note was the focus of reviews on DSTB-associated stigma in health facilities. As of yet, no literature review summarizing the stigma surrounding DRTB among health workers has been found.

Addressing the multiple facets of stigma from national to facility levels is important for a sustainable response to stigma. At the facility level, there is increasing recognition to target the primary factors—the drivers and facilitators—that constitute the stigmatization process. Akin to stigma-reduction interventions for patients and their families that primarily result from an understanding of the factors contributing to the stigma process, DRTB stigma–reduction interventions for health workers likewise require critical analysis of what drives and facilitates the stigmatization. However, unlike patients and their families, where beliefs and lack of knowledge of the disease are common stigma factors, the stigma among health workers could be originated from a range of other factors. Such factors could include innate disease characteristics (eg, being a potent version of TB), and certain features from within the health facilities (eg, institutional policies) and from individual health workers (eg, attitudes and behaviors) [25]. Without an analytic study, intervention reduction efforts for health workers could be undermined.

This scoping review is part of a bigger project that will critically investigate stigma among health workers delivering services to patients resistant to TB medications in the Philippines. This study will provide an overview of the health workers’ stigma surrounding DRTB by explicitly examining the available literature regarding drivers and facilitators contributing to disease stigma, and how it is currently addressed. The findings of this review will offer insights that could help inform appropriate responses toward stigma-reduction interventions for these health workers. Moreover, being part of a bigger project, this review’s findings could also be valuable in the translation and implementation of stigma-reduction measures.

A limitation of this review is its inclusivity in time, language, and place of study; thus, it will only capture part of the picture regarding the stigma associated with DRTB and health workers. Also, the methodological quality of the searched literature will not be assessed. However, the results of this review will inform knowledge users, researchers, DRTB program managers, and implementers to identify key factors leading to stigma among health care workers. The findings of this scoping review will be disseminated through conference and webinar presentations and peer-reviewed journal publications. Should there be modifications to the protocol after its publication, we will provide the details and rationale for the changes, including the dates.

### Conclusions

This review will provide an outline of aspects of DRTB-related stigma confronting health workers. The findings of this review could help inform appropriate responses toward stigma-reduction interventions for these health workers. This is significant because interventions addressing related TB (and DRTB) stigma in the workplace are lacking and such interventions are likely to have a positive impact on both patient care and patient outcomes.

## Appendix

Multimedia Appendix 1 DRTB scoping. DRTB: Drug-resistant tuberculosis.

## Abbreviations

DRTB: drug-resistant tuberculosis
DSTB: drug-susceptible tuberculosis
JBI SUMARI: Joanna Briggs Institute System for the Unified Management, Assessment, and Review of Information
OSF: Open Science Framework
TB: tuberculosis

## Data Availability

All data sets generated and analyzed in this current study will be made available on the Open Science Framework (OSF) website.
