# Supplementary material for: Examining Drug-Resistant Tuberculosis Stigma Among Health Care Workers Toward the Development of a Stigma-Reduction Intervention: Protocol for a Scoping Review
Source: JMIR Res Protoc. 2023 Jan 13;12:e43084. doi: 10.2196/43084 (PMC9883742; doi:10.2196/43084)
Supplement: Multimedia Appendix 1 [file resprot_v12i1e43084_app1.docx]

**Multimedia Appendix 1**

| Table S1: World Health Organization list of high TB- and high DRTB-burden countries | | |
| --- | --- | --- |
| 30 High TB burden countries | |  |
| Angola | Philippines |  |
| Bangladesh | Russia |  |
| Brazil | South Africa |  |
| China | Thailand |  |
| DPR Korea | UR Tanzania |  |
| DR Congo | Vietnam |  |
| Ethiopia | Cambodia |  |
| India | Central African Republic |  |
| Indonesia | Congo |  |
| Kenya | Lesotho |  |
| Mozambique | Liberia |  |
| Myanmar | Namibia |  |
| Nigeria | Papua New Guinea |  |
| Pakistan | Sierra Leone |  |
| Zimbabwe | Zambia |  |
| 30 high drug-resistant TB countries | |  |
| Bangaladesh | Philippines |  |
| China | Russia |  |
| DPR Korea | South Africa |  |
| DR Congo | Thailand |  |
| Ethiopia | Ukraine |  |
| India | Uzbekistan |  |
| Indonesia | Vietnam |  |
| Kazakhstan | Angola |  |
| Kenya | Azerbaijan |  |
| Mozambique | Belarus |  |
| Myanmar | Krygyzstan |  |
| Nigeria | Papua New Guinea |  |
| Pakistan | Peru |  |
| Reprubic of Moldova | Somalia |  |
| Zimbabwe | Tajikistan |  |

Source: WHO. Tuberculosis: World Health Organizarion. <https://www.who.int/health-topics/tuberculosis>. [Accessed on January 15, 2021].

| Table S2. Preliminary Data Base Search Strategies File | |
| --- | --- |
| 1. Insert name of database:   Search conducted on:  Interface  Search Screen  Database  Search term  Limiters  Expanders  Search modes  Number of records retrieved by the search | EBSCOhost  22 September 2022  EBSCOhost Research Databases  Advanced Search  Academic Search Ultimate  drug-resistant tuberculosis OR drug resistant tb OR multidrug-resistant tuberculosis OR multidrug-resistant TB AND (stigma or prejudice or attitude or discrimination) OR (stigmatization or stigmatisation) AND healthcare professionals OR healthcare workers OR ( medical workers or medical staff or health care worker or healthcare employees )  Published Date: 20100101-20220931; Document Type: Abstract; Language: English  Apply equivalent subjects  Boolean/Phrase  379 articles |
| 1. Insert name of database   Search conducted on  Interface  Search Screen  Search term  Expanders  Search modes  Number of records retrieved by the search | Scopus  22 September 2022  <https://www-scopus-com.ezproxy.usq.edu.au/search/form>.  Advanced Search  ( TITLE-ABS-KEY ( drug-resistant AND tuberculosis ) OR TITLE-ABS-KEY ( drug-resistant AND tb ) OR TITLE-ABS-KEY ( multidrug-resistant AND tuberculosis ) OR TITLE-ABS-KEY ( multidrug-resistant AND tb ) AND TITLE-ABS-KEY ( stigma ) OR TITLE-ABS-KEY ( stigmatisation ) OR TITLE-ABS-KEY ( stigmatization ) OR TITLE-ABS-KEY ( stigmatise ) OR TITLE-ABS-KEY ( prejudice ) OR TITLE-ABS-KEY ( attitude ) OR TITLE-ABS-KEY ( discrimination ) AND TITLE-ABS-KEY ( health AND workers ) OR TITLE-ABS-KEY ( health AND professionals ) OR TITLE-ABS-KEY ( medical AND workers ) OR TITLE-ABS-KEY ( medical AND professional ) ) AND PUBYEAR > 2009  Apply equivalent subjects  Boolean/Phrase  108 articles |

| Table S3: Data extraction chart for the scoping review in mapping the evidence of drug-resistant tuberculosis related stigma among health workers in high TB- and drug-resistant-TB burden countries | | |
| --- | --- | --- |
| Key Domain | Sub-domain | Description |
| Citation details | Name of Authors | Indicate the name of the authors |
|  | Year of Publication | Indicate the year when the article was published |
|  | Title of Publication | Indicate the full title of the article |
|  | DOI | Indicate, if provided |
| Language |  | Only English language |
| Type of publication |  | Identifies the article if original review, or grey |
| Source Title |  | Identifies the name of the journal |
| Study details | Aims/Objectives | Describe the aims and objected stated in the study |
|  | Study location | Indicate the study sites (country or region) |
|  | Sample characteristics | Indicate the group of health workers involved in the study |
|  | Sample size | Indicate the number of participants in the study |
|  | Study design | Indicate the methodology adopted e.g. qualitative, quantitative, mixed-method, case study, review. |
|  | Data collection | Indicate the type of data (primary or secondary) and how the data was collected (e.g. interview, survey, etc.) |
|  | Key themes or type of stigma assessed | Indicate the stigma domain (e.g. fear), if primarily identified in the title or objectives of the study |
|  | Description of stigma association | Describe the association of stigma in the finding of the study |
|  | Significant mediators/moderators | Indicate of interventions were identified in the study |
|  | Key findings | Describe the main results (evidence, concept, themes) and link to the scoping review question and objectives |
|  | Study limitations | Indicate the limitations of the study |
| Abstract |  | A copy of the study abstract |
